# Massive Pulmonary Hemorrhage in a Patient With Multiple Pulmonary Cavitary Lesions: A Case Report and Literature Review

**DOI:** 10.7759/cureus.77787

**Published:** 2025-01-21

**Authors:** Ariana S Najera, Matthew Fulton, Nils P Nickel, Gregory Patek, Max Tudela

**Affiliations:** 1 Department of Emergency Medicine, Texas Tech University Health Sciences Center, El Paso, USA; 2 Department of Internal Medicine and Pulmonary and Critical Care, Texas Tech University Health Sciences Center, El Paso, USA; 3 Department of Radiology, Rutgers Health New Jersey Medical School, New Brunswick, USA

**Keywords:** echinococcus, massive hemoptysis, massive pulmonary hemorrhage, multiple pulmonary cavitations, pulmonary cavitations, pulmonary echinococcosis

## Abstract

In this article, we present a rare case of a patient who presented with multiple pulmonary cavitary lesions. The hospital course was complicated by massive pulmonary hemorrhage and subsequent cardiac arrest, during which return of spontaneous circulation was not achieved. Bronchoalveolar lavage (BAL) cultures taken during the hospital stay only resulted positive for *Candida albicans *posthumously. Blood cultures, sputum cultures, and remaining BAL cultures were negative.

This is a rare case of multiple pulmonary cavitary lesions in the setting of a non-immunocompromised patient without evidence of precipitating disseminated disease. Pulmonary echinococcosis is proposed as a possible differential diagnosis in this patient based on clinical, laboratory, and imaging findings.

## Introduction

Pulmonary cavitary lesions are defined as a gas-filled space within a zone of pulmonary consolidation or within a mass or nodule, produced by the expulsion of a necrotic part of the lesion via the bronchial tree. The differential diagnosis of this finding is broad and can include but is not limited to abscess, ischemic necrosis due to malignancy or infarction, and caseous necrosis secondary to tuberculosis (TB). Several bacterial pathogens can form these lesions, the most common of which include TB, *Klebsiella*, and *Staphylococcus*. Patients presenting with multiple rather than solitary lesions require an extensive workup for multiple pathogens, of which the most common culprits are TB, septic emboli, invasive aspergillosis, and coccidioidomycosis. Inflammatory causes should also be considered, such as granulomatosis with polyangiitis and rheumatoid nodules [1]. Parasitic infections can also cause multiple cavitary lesions in patients by the displacement of lung tissue by cystic structures. *Echinococcus* is a cestode tapeworm that is clinically relevant in humans for its ability to cause devastating infection. Two species that can spread to the lungs include *Echinococcus granulosus* and *Echinococcus multilocularis*; they have the ability to spread hematologically into several organ systems and cause pulmonary disease. The most commonly affected organ is the liver, followed by pulmonary spread in up to a third of cases. Many human carriers can be asymptomatic, while those who develop pulmonary lesions are likely to cause symptomatic disease [2].

The case involves a 41-year-old male patient who initially presented with multiple pulmonary cavitary lesions. Despite a negative workup throughout his hospital stay, he subsequently suffered from massive hemoptysis followed by cardiac arrest and was unable to be resuscitated. The differential diagnoses for this patient were broad and included TB, fungal infections, atypical pneumonia, malignancy, septic emboli, and vasculitis. Interferon-gamma release assay (IGRA), beta-D-glucan assay, and sputum cultures all returned negative, effectively ruling out TB, *Aspergillus*, and pneumonia as potential causes. The bronchoalveolar lavage (BAL) cultures resulted positive for *Candida*, but negative for phagocytized organisms, which is favorable for contaminants rather than true *Candida* infection. *Candida* species exist as normal flora of the human skin, oropharynx, lower gastrointestinal tract, and genitourinary system, and colonized patients will often test positive without clinical relevance. Respiratory cultures are unreliable for the diagnosis of invasive candidiasis because the organism often colonizes the upper airways. A definitive diagnosis requires culturing *Candida* from blood. In this case, *Candida* is an unlikely cause of this patient’s cavitary lung lesions and clinical presentation since he had no evidence of disseminated candidiasis (negative blood cultures), which most often precipitates pulmonary candidiasis [3]. The patient was also worked up for vasculitis; however, a negative anti-neutrophil cytoplasmic antibody (ANCA) IgG made vasculitis an unlikely diagnosis. Anti-glomerular basement membrane (anti-GBM) antibodies, a marker for Goodpasture syndrome, were not obtained during the workup. However, non-specific markers such as anti-nuclear antibodies (ANAs) were negative as well, and Goodpasture syndrome was unlikely in this case because the patient did not have evidence of kidney injury. An echocardiogram also showed no evidence of vegetation, and negative blood cultures made infectious endocarditis an unlikely consideration. Hepatitis panel, respiratory pathogen panel test, and human immunodeficiency virus (HIV) were also negative. No consideration was given to surgical or transbronchial biopsy, as it is not standard of care for a case like this, especially in the light of clinical deterioration.

Unfortunately, a definitive diagnosis was unable to be established for this patient prior to his passing away. However, based on all the findings during this patient's workup as further described in this case report, echinococcosis is a strongly considered differential diagnosis and will be the focus of this case report and will be discussed in further detail.

## Case presentation

A 41-year-old male patient presented to the Emergency Department (ED) with a 10-day history of progressively worsening weakness and cough. The patient was previously admitted to a hospital in Juarez, Mexico, where he was diagnosed with diabetic ketoacidosis (DKA), sepsis secondary to pneumonia, and influenza. The patient was discharged from that hospital after a three-day stay the same day he presented to the ED. The patient presented due to symptoms not improving after his previous hospital stay. His symptoms initially began with a sore throat and rhinorrhea that eventually progressed to fever, weakness, cough, and intermittent bloody sputum. The patient also noted recent unintentional weight loss. The patient denied chills, chest pain, shortness of breath, nausea, vomiting, diarrhea, or sick contacts. He did admit to purchasing 40 chickens approximately 20 days prior, which he had daily contact with. None of his close contacts exhibited similar symptoms. The patient had a history of medication non-compliant hypertension and type 2 diabetes mellitus and denied a history of smoking, alcohol consumption, IV drug use, home medications, or surgical history. The patient resided in Juarez, Mexico. Upon physical exam, the patient was alert and oriented, appeared well nourished, had no obvious respiratory distress, and was able to speak in full sentences. Diffuse rhonchi were noted on auscultation. Oxygen saturation was 91% on room air, improved to between 95% and 97% on 2 L via nasal cannula. The initial heart rate was around 120 beats per minute. The remainder of the physical exam was unremarkable.

**Figure 1:**
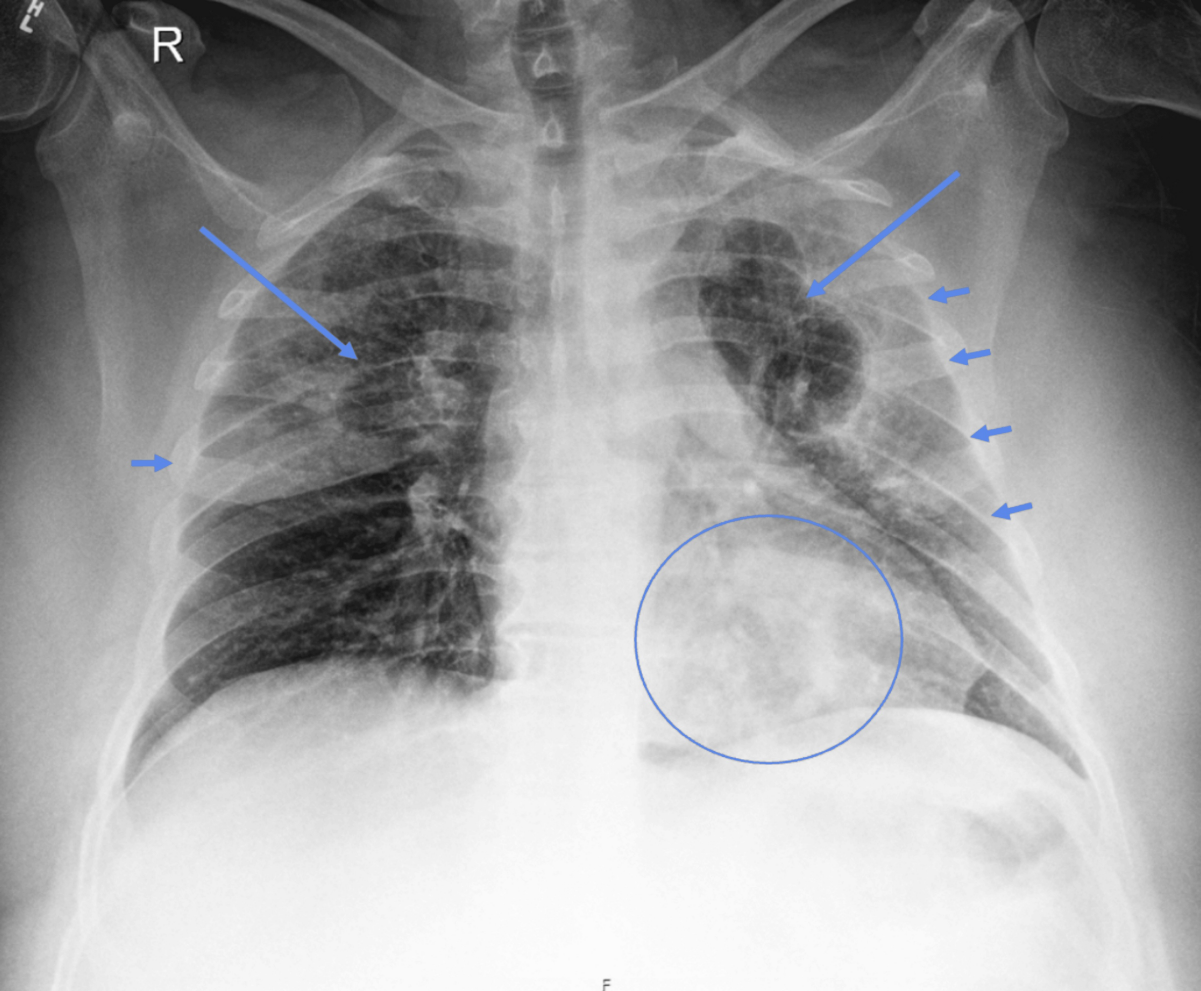
AP chest radiograph shows bilateral upper lobe predominant hazy opacities with a more confluent opacity lining the minor fissure and the left lateral pleura (arrowheads). Two cavitary lesions are noted in the upper lobes (arrows). There is a subtle third left lower lobe retrocardiac lesion.

**Figure 2:**
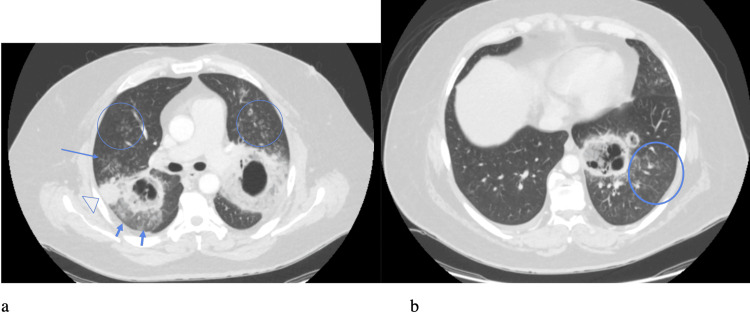
Axial contrast-enhanced CT scan with lung windows (a) shows bilateral upper lobe spiculated thick-walled cavitary lesions with surrounding ground-glass opacification (arrowheads) suggestive of parenchymal involvement. Also noted are adjacent tree-in-bud nodules and diffusely scattered random nodules (circles), representing diffuse spread of disease. An adjacent subpleural consolidation is also visualized in the right upper lobe (open arrowhead). Axial contrast-enhanced CT at another level (b) shows a left lower lobe retrocardiac consolidative lesion with multiple internal cavities suggesting areas of necrosis, also with surrounding pulmonary nodularity.

**Figure 3:**
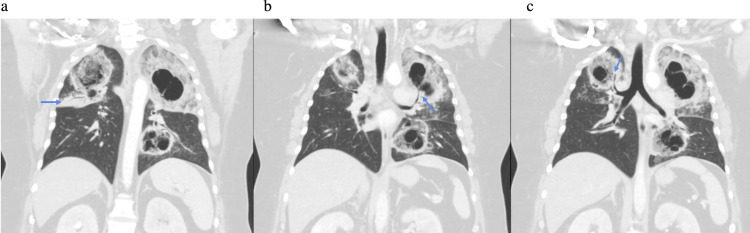
Coronal reformats again show the multiple cavitary lesions in the bilateral upper lobes and left lower lobe. There is a right upper lobe consolidation with air bronchograms about the minor fissure, corresponding to the confluent opacity seen on chest X-ray (arrow in a). Note the direct connection of the cavitary lesions with the bronchial tree demonstrated in the left upper lobe (arrow in b) and the right upper lobe (arrow in c), which corresponds with direct bronchial spread of disease.

Initial chest X-ray demonstrated bilateral upper lung hazy and confluent airspace opacities with possible cavitation suggested (Figure 1). CT chest was obtained to evaluate further, which demonstrated cavitary lesions involving the bilateral upper lobes and left lower lobe (LLL) with surrounding airspace consolidation, measuring up to 10.3 in the left upper lobe (LUL) (Figures 2, 3). Peribronchovascular airspace opacities were adjacent to the cavitary lesions. These findings raised concerns about infectious processes including bacterial, mycobacterial, or fungal infection. The patient was started on broad-spectrum antibiotics vancomycin and piperacillin/tazobactam empirically. He was then admitted to Internal Medicine for further workup and management.

During his hospital stay, he was being worked up for necrotizing pneumonia with cavitary lesions in the presence of neutrophilic leukocytosis. Differential diagnosis was broad and included TB, fungal infections, atypical pneumonia, malignancy, septic emboli, and vasculitis. Hepatitis panel, blood cultures, respiratory pathogen panel test, HIV 1/2 immunoassay, ANCA IgG, IGRA, and beta-D-glucan were negative. Sputum cultures were negative for acid-fast bacteria and fungi and demonstrated normal oral bacterial flora. Blood cultures remained negative throughout his hospital course. The patient was started on piperacillin/tazobactam and vancomycin and required supplemental oxygen at 3 L per minute via nasal cannula. Elevated liver enzymes were also present. Abdominal ultrasound was obtained to further evaluate, which demonstrated an echogenic liver suggesting mild fatty infiltration without evidence of liver cysts or abscesses.

**Figure 4:**
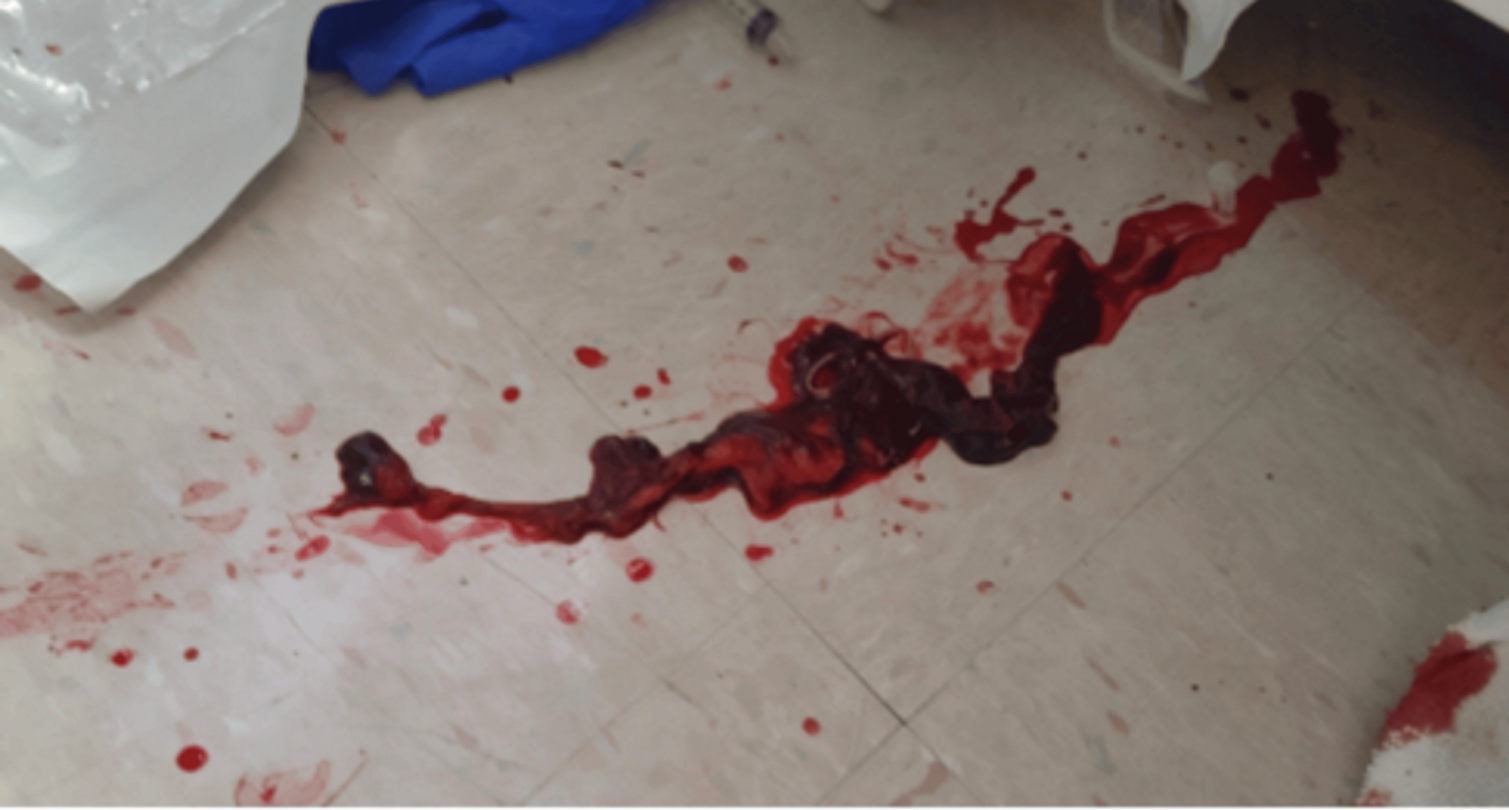
Blood clot evacuated from the patient’s oral cavity during intubation.

On hospital day two, piperacillin/tazobactam and vancomycin were discontinued, and the patient was started on ceftriaxone and azithromycin. Pulmonology was consulted for bronchoscopy with BAL for further workup of cavitary lesions. The patient consented to further HIV workup consisting of CD4/CD8 and viral load HIV, which showed normal CD4/CD8 counts and negative HIV. On hospital day three, the pulmonologist saw the patient and scheduled a bronchoscopy for the following day. The antibiotic regimen was changed to ampicillin/sulbactam 3g IV Q6H. Azithromycin was continued. On hospital day four, a bronchoscopy was performed with BAL in the right upper lobe (RUL), LUL, and LLL, and findings included thick, purulent secretions in all segments of the lung as well as purulent secretions in the right main stem. Bronchial mucosa and anatomy in the left lung were normal without endobronchial lesions. The return fluid in the RUL and LUL was clear, and the return fluid in the LLL was purulent. Upon examination of the patient after the procedure, saturation was 93% on a simple face mask at 7 L per minute. The patient reported an increase in cough frequency after the procedure. He developed low-grade fevers and was started on external cooling measures and oral acetaminophen. On hospital day five, the patient reported improvement in symptoms from the previous day with a noted decrease in cough frequency. He remained on 7 L with a simple face mask with appropriate saturations in the mid-90s. The patient remained on azithromycin and ampicillin/sulbactam IV. On the night of hospital day five, a rapid response was called for severe, massive hemoptysis. Upon the physician entering the room, the nurses had evacuated approximately 800 cc of blood from the oral cavity, which was collected in a bedside suction canister. The patient was found to be lethargic, poorly responsive, pale, and diaphoretic, and an oxygen saturation was unable to be obtained. He was immediately intubated for airway protection, and during the laryngoscopy procedure, a large clot measuring approximately 2.5 cm in width and 24 cm in length was physically removed through the oral cavity due to the inability to suction (Figure 4). Soon after, the patient became unresponsive and pulseless, and cardiopulmonary resuscitation (CPR) was initiated. The patient underwent a total of 11 cycles of CPR; the rhythm was persistently asystolic. Bedside point-of-care ultrasound (POCUS) was performed; no acute pathologies such as pleural effusion, pericardial effusion, or pneumothorax were detected; and there was no cardiac activity appreciated. The patient was subsequently pronounced deceased. Posthumously, the BAL resulted positive for *Candida albicans*; all other cultures remained negative. Unfortunately, no autopsy was performed, and the patient was subsequently cremated.

## Discussion

In this case report, we described a patient who presented with multiple, bilateral pulmonary cavitations of unknown origin and cause. The hospital workup was negative except for *C. albicans* on BAL culture. Tragically, the patient succumbed to a massive pulmonary hemorrhage before additional workup could be done to further evaluate the cause of this presentation. Since the BAL cultures were positive for *C. albicans*, a diagnosis of pulmonary candidiasis was considered but deemed unlikely. This is in part due to *Candida* being a common respiratory tract and oral flora and commonly colonized without causing disease. *Candida* species colonize the respiratory tract in approximately half of all healthy individuals [4]. In addition, this patient had no signs of disseminated candidiasis, which is the most common cause of pulmonary candidiasis [3].

The imaging findings of isolated pulmonary candidiasis are most often in the form of diffuse or lobar bronchopneumonia or multiple pulmonary nodules [5]. In the literature review, only one case report was found that described a patient with multiple cavitary lesions in the setting of pulmonary candidiasis; however, this patient had positive blood cultures for *Candida* species, which our patient did not [6]. The differential for the imaging findings in this case is broad, given that pulmonary cavitary lesions are not specific to a single disease process. The involvement of the bronchial tree can be seen in multiple disease processes including metastatic cancer, fungal infections, and bacterial pneumonia. This patient had an extensive workup for both fungal and bacterial causes, which resulted negative. However, a potential diagnosis not previously considered during the patient’s hospital stay can be considered-pulmonary echinococcosis.

It is estimated that approximately one million people are infected with *Echinococcus* at any one time [7]. Echinococcosis is a parasitic disease that occurs in the larval stages of *Echinococcus* cestodes. The most common manifestation in humans is cystic echinococcosis, which most often occurs in the liver, and the second most common site is the lungs (25%) [8]. Echinococcosis is very rare in the United States but is an endemic disease in South America, Mediterranean countries, Australia, parts of Africa, and Asia. In an infected individual, echinococcosis occurs after the eggs hatch and larvae are released in the small intestine. The mucosa in the small intestine is invaded, and the larvae are spread through the bloodstream and deposited in various organs such as the liver or lungs [9]. Once larvae enter the organ, they start gradually transforming into hydatid cysts. Humans can acquire the infection through ingesting uncooked vegetables, drinking infected water, or handling contaminated dirt or animal hair. Cough is the most common presentation of pulmonary echinococcosis, as well as chest pain, dyspnea, fever, and hemoptysis, all of which our patient presented with. Our patient’s symptoms were consistent with echinococcosis, and hydatid cysts have the ability to erode major vessels such as the aorta or pulmonary arteries and could cause massive hemoptysis [10].

Human echinococcosis in Mexico is rare and usually involves immigrants or travelers. Mexican-acquired human echinococcosis is rarely reported and tends to occur in rural environments with poor access to modern water infrastructure, exposure to farm animals, contaminated vegetables, and poor hygiene [11]. Reported veterinary infections tend to occur in central and southern Mexico; there has been discussion that cases in Mexico may be under-diagnosed and under-reported; however, the few epidemiological studies that have been conducted on this topic support low prevalence [12-14].

Radiographic features of pulmonary echinococcosis infections can be variable and range from smooth, well-defined fluid-filled homogenous lesions to complicated cysts with contained or complete rupture. Cyst rupture is the most common complication of pulmonary hydatid disease [15]. On CT scans, several different characteristics of cyst rupture have been described, including water lily, crescent, serpent, spin, cumbo, and mass-within-cavity signs [16-18]. In a study involving 72 patients with pulmonary hydatid cysts, those with complicated cysts were more likely to exhibit hemoptysis and diffuse pulmonary infiltrates rather than well-defined nodular cysts [19]. The patient in this case exhibited some radiographic features suggestive of complicated hydatid cyst disease, including the cumbo sign, also known as onion peel or double arch sign [16]. This is evident as air-fluid levels within the cystic cavity, suggestive of endocyst shrinkage and rupture due to the increasing amount of air [16]. Hydatid cysts can rupture into bronchi and blood vessels and also into the surrounding lung parenchyma. This will appear on imaging as centrilobular opacities around the cystic cavity, which can be appreciated in the imaging obtained from our patient [16]. Importantly, none of these radiographic findings alone are diagnostic for pulmonary echinococcosis [10]. The consolidations and ground-glass opacities surrounding the cavitary lesions can be seen with hemorrhage, inflammation, or necrosis.

Pulmonary echinococcosis is typically diagnosed using a combination of imaging findings, clinical suspicion, and laboratory studies. Serology can be used to aid in the diagnosis of this disease, with the most sensitive test being *Echinococcus* IgG enzyme-linked immunosorbent assay (ELISA), with 94% sensitivity [20]. It is very important to note, however, that negative serology testing does not rule out disease, especially in cases of isolated, unruptured pulmonary cysts. One study found that only 12.5% of patients with isolated, unruptured pulmonary echinococcosis had a positive IgG test. It was also noted that the sensitivity of the IgG ELISA for pulmonary echinococcosis is lower than that in the liver [21]. In cases of suspected echinococcosis with negative serology tests, cyst aspiration or biopsy can be utilized [22]. Other supporting laboratory findings include anemia, eosinophilia, and elevated liver function testing. Our patient presented with anemia and elevated liver enzymes but did not have eosinophilia. This is not an absolute finding in echinococcosis, and one study from India found eosinophilia in only 42.3% of patients [23]. Treatment of pulmonary echinococcosis depends on the appearance of the cysts themselves and whether there are calcifications. In most cases, the treatment consists of albendazole at a minimum and plus or minus PAIR, which is puncture, aspiration, injection of 95% ethanol solution or hypertonic saline solution, and re-aspiration [24]. Surgery is the treatment of choice for cysts that have ruptured if they are compressing vital structures or causing hemorrhage or secondary infection [20,25,26].

Based on a combination of the CT findings, laboratory findings, and clinical presentation, we are considering pulmonary echinococcosis to be a potential differential diagnosis for this patient. Although this remains unconfirmed, this case was a demonstration of interesting clinical findings with a negative workup, and retrospectively, echinococcosis should have been considered as a differential diagnosis for this patient.

This case report, although with an unverified diagnosis, serves an educational purpose by highlighting echinococcosis as a potential differential in areas close to endemic regions, especially border towns. This diagnosis is rare but should be considered in patients who have immigrated from endemic areas or patients who live in border town areas or recently traveled to endemic areas. We hope that this case report will aid in differential diagnoses for patients who present with cavitary lesions with otherwise negative workups, and we hope that this diagnosis may be considered earlier and, thus, treatment initiated earlier, perhaps even while the patient is still in the ED.

## Conclusions

Cystic echinococcosis is a public health problem, affecting mostly rural regions in developing countries. In most developed countries, it is found almost exclusively in migrants from endemic regions. However, we need to maintain a high level of suspicion of this disease especially in border towns across the United States, since those areas are more affected by diseases not endemic to this country. Although an uncommon disease in the United States, if we are faced with a patient with suspicious pulmonary or hepatic lesions with an overall negative workup for more common diseases, we should be utilizing *Echinococcus* IgG ELISA testing in these patients and still have high clinical suspicion even if the serology testing is negative. If left untreated, it can have devastating consequences as seen in the patient presented in this case report. As noted previously, echinococcosis was not confirmed as being the causative disease for this patient; however, based on the clinical, laboratory, and imaging findings, it remains a possible diagnosis.
